# The prevalence of CTNNB1 mutations in primary aldosteronism and consequences for clinical outcomes

**DOI:** 10.1038/srep39121

**Published:** 2017-01-19

**Authors:** Vin-Cent Wu, Shuo-Meng Wang, Shih-Chieh Jeff Chueh, Shao-Yu Yang, Kuo-How Huang, Yen-Hung Lin, Jian-Jhong Wang, Rory Connolly, Ya-Hui Hu, Celso E. Gomez-Sanchez, Kang-Yung Peng, Kwan-Dun Wu

**Affiliations:** 1 Department of Internal Medicine, National Taiwan University Hospital, Taipei, Taiwan; 2 Department of Urology, National Taiwan University Hospital, Taipei, Taiwan; 3 Cleveland Clinic Lerner College of Medicine and Glickman Urological and Kidney Institute, Cleveland Clinic, USA; 4 TAIPAI, Taiwan Primary Aldosteronism investigator, Taipei, Taiwan; 5 Division of Nephrology, Department of Internal Medicine, Chi Mei Medical Center, Liouying, Tainan City, Taiwan; 6 EKF Diagnostics, Cardiff, Wales, UK; 7 Department of internal medicine, Taipei Tzu Chi Hospital, Buddhist Tzu Chi Medical Foundation, Taipei, Taiwan; 8 Division of Endocrinology, G.V. (Sonny) Montgomery VA Medical Center and Department of Medicine, The University of Mississippi Medical Center, Jackson, MS, USA

## Abstract

Constitutive activation of the Wnt pathway/β-catenin signaling may be important in aldosterone-producing adenoma (APA). However, significant gaps remain in our understanding of the prevalence and clinical outcomes after adrenalectomy in APA patients harboring *CTNNB1* mutations. The molecular expression of CYP11B2 and gonadal receptors in adenomas were also explored. Adenomas from 219 APA patients (95 men; 44.2%; aged 50.5 ± 11.9 years) showed a high rate of somatic mutations (n = 128, 58.4%). The majority of them harbored *KCNJ5* mutations (n = 116, 52.9%); 8 patients (3.7%, 6 women) had *CTNNB1* mutations. Patients with APAs harboring *CTNNB1* mutations were older and had shorter duration of hypertension. After adrenalectomy, *CTNNB1* mutation carriers had a higher possibility (87.5%) of residual hypertension than other APA patients. APAs harboring *CTNNB1* mutations have heterogeneous staining of β-catenin and variable expression of gonadal receptors and both CYP11B1 and CYP11B2. This suggests that *CTNNB1* mutations may be more related to tumorigenesis rather than excessive aldosterone production.

Primary aldosteronism (PA), which is characterized by hyperaldosteronism, affects 20% of patients with resistant hypertension^1^. Somatic mutations in the selectivity filter of the potassium channel, GIRK4 (encoded by *KCNJ5*) in aldosterone-producing adenoma (APA) result in a loss of potassium selectivity and entry of sodium and membrane depolarization^2^. Further somatic mutations in a subunit of an L-type voltage-gated Ca^2+^-channel, Cav1.3 (encoded by *CACNA1D*) and in 2 ATPases (Na^+^/K^+^-ATPase alpha subunit and Ca^2+^-ATPase 3, encoded by *ATP1A1* and *ATP2B3*, respectively) have also been identified^3^,^4^,^5^. The resultant Ca^2+^ influx and activation of the Ca^2+^ signaling pathway leads to the increase in *CYP11B2* gene transcription and an increase in aldosterone biosynthesis^2^. *CTNNB1* (β-catenin) mutations have been reported in APAs^6^ and in cortisol-secreting or non-functional adenomas^7^. Constitutive activation of β-catenin in the adrenal cortex of transgenic mice resulted in progressive steroidogenesis, adrenal hyperplasia and late development of malignant characteristics and excessive secretion of aldosterone^8^. Moreover, overexpression of β-catenin in adrenal cortical carcinoma (ACC) has been correlated with a worse prognosis^9^. Recently, cases of APAs harboring activating mutations of β-catenin, which expressed high levels of the gonadal receptors LHCGR and GNRHR, were described in three women (two during pregnancy and one postmenopausal) where Wnt activation caused adrenocortical cells to de-differentiate toward an adrenal-gonadal precursor cell^10^. The aim of this study was to determine the prevalence of the *CTNNB1* mutations in APA patients and to correlate the mutation status with clinical outcomes in order to determine the outcomes on patients who harbor these mutations.

## Materials and Methods

### Ethics Declaration

This study has been approved, supervised and monitored by the institutional review board of National Taiwan University Hospital, Taipei, Taiwan (No. 200611031 R). It complied with the Declaration of Helsinki. All participants signed the informed consent before they were included in the study.

### PA Identification

The present study was based on the Taiwan Primary Aldosteronism Investigation (TAIPAI) database and tissue bank^11^,^12^,^13^. The TAIPAI database was constructed from June 2008 to March 2011 for quality assurance, including two medical centers, three affiliated hospitals and 2 regional hospitals in different cities in Taiwan^14^. All antihypertensive medications were discontinued for at least 21 days before confirmatory and lateralizing tests. Doxazosin and/or diltiazem were administered to control markedly high blood pressure where required^15^.

The diagnosis and subtype identification of PA were established and performed according to the standard protocol of TAIPAI, including saline infusion test, adrenal venous sampling and NP-59 scintigraphy with SPECT-CT imaging^11^,^12^,^13^,^16^ (supplementary file and Figure S1).

### Adrenalectomy

The adrenalectomy was performed via the lateral trans-peritoneal laparoscopic approach by experienced surgeons, and adrenal tumors removed during the surgery were freshly frozen and stored at −80 °C.^17^.

### Sequencing

#### Nucleic acid extraction

Genomic DNA was extracted from 219 paired adenoma and its peritumoral normal adrenal cortices. Tumor DNA was extracted via a QIAamp DNA mini kit (Qiagen, Hilden, Germany); total RNA was isolated from frozen tissue using Trizol (Invitrogen, Carlsbad, Ca, USA) and then cleaned-up by using the GENEzol TriRNA Pure Kit (Geneaid, New Taipei City, Taiwan). After DNaseI treatment (Invitrogen, Carlsbad, Ca, USA), 500 ng of total RNA was reverse-transcribed using Moloney Murine Leukemia Virus Reverse Transcriptase (M-MLV RT, Promega, Madison, WI, USA) and random hexamers (Promega, Madison, WI, USA) according to the manufacturer’s instructions. Relative gene expression in relation to GAPDH was calculated with the formula: 2^*-(Ct of target gene-Ct of* GAPDH)^.

#### Sequencing of somatic mutations

The coding area of the genomic DNA was investigated by exon sequencing. The entire coding sequence and flanking regions of *candidate mutations* were amplified and sequenced using gene-specific primers as previously reported^18^. Accordingly, the PCR primers used to amplify fragments for direct sequencing of *CTNNB1/ATP1A1/ATP2B3/CTNNB1* and *CACNA1D* also followed previous reports^3^,^5^,^10^,^19^,^20^ (listed in supplement Table S1). The annealing temperature was 58 °C. Direct sequencing of PCR products was performed using The BigDye^®^ Terminator v3.1 Cycle Sequencing Kit (Applied Biosystems, Foster City, USA) with a 3730 DNA Analyzer (Applied Biosystems, Foster City, USA). Patients who were diagnosed with family type I (FH-I)/glucoticoid remediable aldosteronism (GRA) were identified via long-range polymerase chain reaction as described previously^21^ (Table S1).

#### Tissue Immunohistochemistry

Immunohistochemistry (IHC) was performed using mouse monoclonal antibody for *CYP11B2* and rat monoclonal antibody for *CYP11B1* (a kind gift from Professor Celso Gomez-Sanchez^22^). Commercial antibodies to β-catenin (05–665, Millipore), GnRHR (ab183079, Abcam), LHCGR (GTX100008, GeneTex), and GATA4 (GTX113194, GeneTex) were also used. The sections of paraffin- embedded adrenal tumor and surrounding tissues for IHC were stained using the non-biotin-amplified method (Novolink; Novocastra Laboratories) according to the manufacturers’ protocol. Images were acquired with fluorescence microscope Olympus BX51 combined with Olympus DP72 camera and cellSens Standard 1.14 software (Olympus, Germany). The sections were quantified at 40× and 400× magnification.

#### Western blotting analysis

Human adrenal specimens were homogenized in T-PER tissue protein extraction reagent (Thermo-Fisher) containing a protease inhibitor cocktail (Roche). A 30 μg sample of protein from each specimen was separated using SDS-PAGE and transferred onto PVDF membranes (Millipore). Visinin-like 1 (VSNL1) is upregulated in aldosterone-producing adenomas (APAs) compared with normal adrenals^23^. *CTNNB1* exon 3 mutations could be involved in APA formation due to accumulation of β-catenin and increased expression of Cyclin D1^24^. The canonical (Wnt/β-catenin-mediated) signaling functionally interacts with GATA4^25^, a marker of gonadal differentiation that is crucial in adrenal development^26^. Therefore, the primary antibodies used were as follows: mouse monoclonal anti–active β-catenin (Anti-ABC) (05–665, Millipore), mouse monoclonal antibody for CYP11B2 (a kind gift from Professor Celso Gomez-Sanchez), rabbit monoclonal anti-Cyclin D1 (ab134175, Abcam), rabbit polyclonal anti–VSNL1 (GTX115039, GeneTex), and rabbit polyclonal anti-GATA4 (GTX113194, GeneTex). Levels of proteins were detected using chemiluminescent detection reagents (Millipore) and visualized using a UVP Biospectrum 810 imaging system (Ultra Violet Products Ltd, Cambridge, UK).

#### Measure of outcomes

For the first 3 months post-operatively the patients were followed monthly, and every three months subsequently. Evaluation of ’cured’ hypertension has been described previously^27^. Hypertension was considered as ‘cured’ if 75% of the recorded systolic BP was < 140 mmHg and the diastolic BP was < 90 mmHg without taking antihypertensive medications at least 1 year after adrenalectomy^27^,^28^.

We further studied the differences in patients who had *CTNNB1, KCNJ5* mutation*s* or no-identified mutation (wild-type; WT), and defined them as the ‘enrolled group’. Given the differences in the baseline characteristics among the ‘enrolled group’ patients during the statistical analysis, we attempted to match each patient in the *CTNNB1* mutation group with 3 patients in the *KCNJ5* mutation group and 3 patients in the WT group (matched group), based on nearest neighbor matching without replacement, using age, sex and mean blood pressure (MBP).

#### Statistical analysis

All data were expressed as the mean ± standard deviation (SD). A *p*-value of < 0.05 was considered significant.

Statistical analyses were performed with R software, version 2.8.1 (Free Software Foundation, Inc., Boston, MA, U.S.A.). Aldosterone and ARR were log-transformed to normal distribution. Logistic regression analysis with a stepwise variable selection procedure was applied using available variables to identify important factors associated with post-operative residual hypertension. The goodness-of-fit (GOF) of the fitted multiple logistic regression model was assessed by the estimated area under the receiver operating characteristic (ROC) curve, the Hosmer-Lemeshow GOF test, and the adjusted generalized *R*^2^.

## Results

### Patient characteristics

#### Demography of patients and distribution of genetic alterations

Among 219 APA patients (95 men; 44.2%) who underwent adrenalectomy, the rate of somatic mutations was 58.4% (n = 128). Targeted sequencing for the reported mutations in APAs of *KCNJ5, CACNA1D, ATP1A1, ATP2B3 and CTNNB1* exons was performed from the adenomas. *CTNNB1* mutations were found in 8 of the 219 patients (3.7%) with APAs, of which there were 3 with S45F and 5 with S45P mutations. The detected mutations occurred in conserved serine/threonine residues in exon 3 (Table 1, Figures s2 ands s3). The absence of *CTNNB1* mutations in all peripheral blood DNA samples and in paired peri-tumoral adrenal cortices confirmed the somatic nature of the genetic alteration.

**Figure 1:**
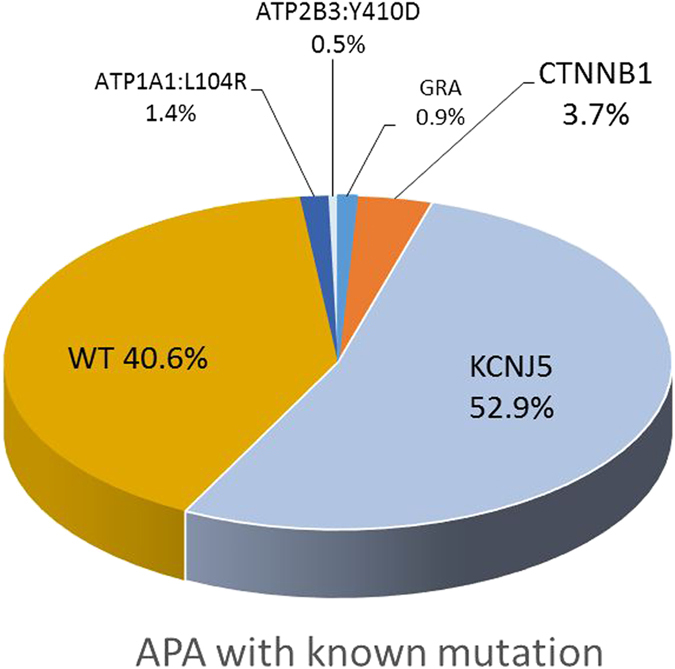
The distribution of known mutation of aldosterone producing adenoma in the cohort (n = 219).

The majority of the somatic mutations were positive for *KCNJ5* mutations (n = 116, 52.9%) (Figure s4). Sequencing of adenoma samples revealed the occurrence of the following somatic *KCNJ5* mutations: p.Gly151Arg (c.451 G > A or c.451 G > C) (n = 64), p.Leu168Arg (c.503 T > G) (n = 48), p.Ile157del (c.470_472delTCA) (n = 1), and p.Thr158Ala(c.472 A > G) (n = 3) mutations in the heterozygous state. (Fig. 1).

Besides the 128 somatic mutations, we also identified that there were two APA patients noted to have germ-line glucocorticoid remediable aldosteronism (GRA) mutations from two different families among the 219 APA patients investigated.

#### Enrolled group and matched group

We further studied the ‘enrolled group’, defined as those patients without mutated adenoma (WT group) and those with *CTNNB1*or *KCNJ5* mutations (n = 213). *CTNNB1* mutation carriers were older (p < 0.001), had higher serum potassium and creatinine levels compared with those with *KCNJ5* mutations. The duration of hypertension is shorter among *CTNNB1* mutation carriers than *KCNJ5* mutation carriers or WT APA patients (all p < 0.001). The majority of patients harboring *CTNNB1* mutations were female (75%, n = 6), however the sex ratio is not significantly different from patients harboring *KCNJ5* mutations or WT. Furthermore, the tumor size and ratio of parental hypertension were not significantly different among patients who had *CTNNB1* mutations, *KCNJ5* mutations or WT (Table 2).

#### Factors predicting post-operative residual hypertension

Hypertension was considered ‘cured’ in 144 (67.6%) of APA patients, defined as taking no antihypertensive agents at one year after adrenalectomy. Most (n = 109, 75.7%) cured patients became normotensive within 6 months after surgery; 27 patients (18.8%) became normotensive after 9 months, and 8 took up to 1 year (6.3%) (Table 3).

Compared with the *KCNJ5* mutation carriers (12.5% vs. 79.3%, *p *< 0.001) and WT group (12.5% vs. 57.3%, *p* = 0.018), the *CTNNB1* mutation carriers had a much lower chance of recovery from hypertension even after one-year follow-up. This result remained the same after matching — *CTNNB1* mutation carriers had a significantly lower cure rate for hypertension (12.5% vs. 66. %, *p* = 0.011 compared with matched *KCNJ5* mutation carriers; 12.5% vs. 54.2%, *p* = 0.047 and with matched WT patients).

Even after adjustment for possible variables when compared with all those who had *KCNJ5* mutations, being a *CTNNB1* mutation carrier was an independent factor to predict post-operative residual hypertension [odds ratio (OR) = 18.9, *p* = 0.010] (Table 2). When the *CTNNB1* mutation carriers were compared with all WT APA patients, the chance of having residual hypertension was not significant (*p* = 0.051). However, after matching for the effects of age, sex and blood pressure, APA patients who had *CTNNB1* mutations had significantly higher risk of post-operative residual hypertension than either *KCNJ5*-mutaion carriers (OR = 18.2, *p* = 0.046) or WT patients (OR = 14.5, *p* = 0.028).

The final multiple logistic regression model had a high discriminatory power and fitted the observed binary data well before matching (adjusted generalized R^2^ = 0.290 and Hosmer-Lemeshow goodness-of-fit (GOF) test *p* = 0.536). After matching, the adjusted generalized R^2^ = 0.564, and Hosmer-Lemeshow GOF test *p* = 0.620 which showed the model fitted with the data.

#### mRNA and protein expressions in investigated APA

**Figure 2:**
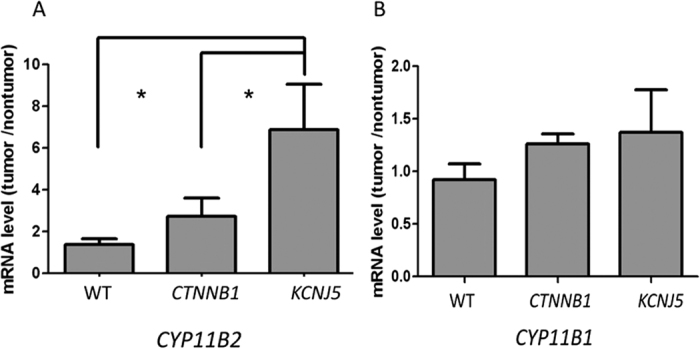
The expression of (**A**) *CYP11B1*, and (**B**) *CYP11B2* in PA patients with *CTNNB1, KCNJ5* mutation and WT. mRNA levels corrected with GAPDH mRNA levels measured by RT-PCR and expressed as tumor/non-tumorous adrenal cortex ratio, *p < 0.05.

**Figure 3:**
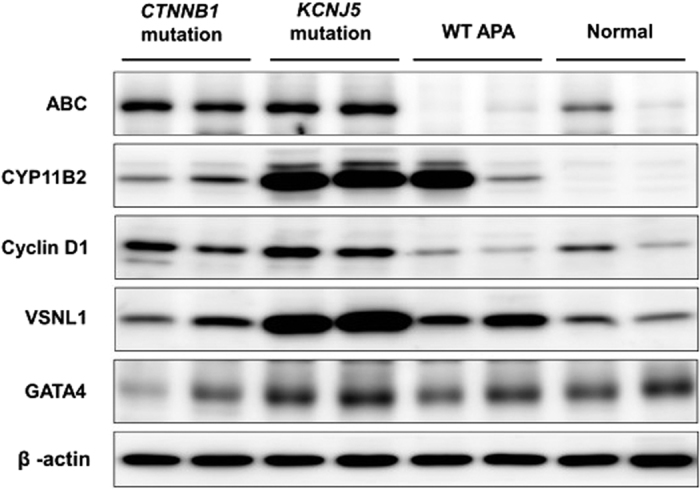
A representative western blot (30 μg/well) for tissue lysate. The expression levels of ABC, CYP11B2, CyclinD1, VSNL1 and GATA4 in adenoma from patients harboring *CTNNB1* mutation, *KCNJ5* mutation, wild type and controlled adrenal gland were determined by western blot analysis. Abbreviations ABC, active β–catenin; APA, aldosterone producing adenoma, VSNL1, Visinin-like 1, WT, wild type.

**Figure 4:**
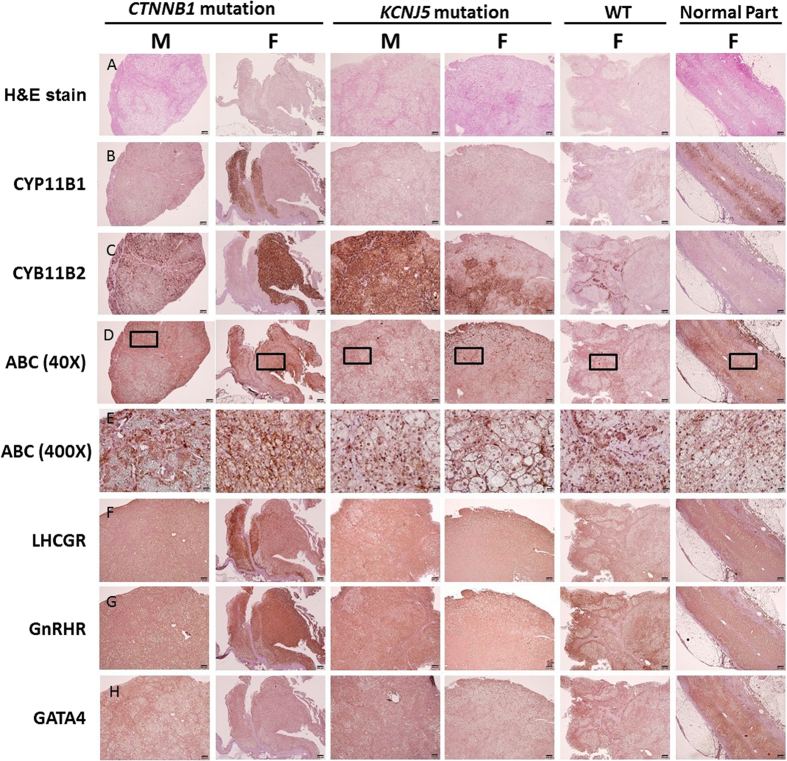
Histologic expression in patients harboring *CTNNB1* and *KCNJ5* mutations and in WT patients. (**A**) H&E stain of investigated adenoma. The WT was from adrenal cortex. (**B**) *CYP11B2* expressed diffusely on adenoma harboring *CTNNB1* mutation and mottled expression on adenoma harboring *KCNJ5* mutation. (**C**) However, CYP11B1 stating showed strong expression at there with low CYP11B2 expression. (**D**) Adenomas displayed weak nuclear and cytoplasmic active β-catenin expression, especially in female patients harboring *CTNNB1* mutation. Bar, 50 mm (X40) and (**E**) in a high magnification view (X400). (**F**) LHGHR was diffusely expressed in the adrenal tissue and was also prominent in adenomas harboring *CTNNB1* mutation. (**G**) GnRHR showed diffuse cytoplasmic, membranous and nuclear expression in adenomas, and was most enhanced in adenomas harboring *CTNNB1* mutation or wild type. (**H**) The tissue expression of GATA4 in adrenal tissue was not significant. Abbreviations ABC, active β–catenin; GnRHR, gonadotropin-releasing hormone receptor, HE, hematoxyline and eosin. LHGHR, luteinizing hormone-chorionic gonadotropin receptor.

The results of mRNA expression from real-time PCR showed that *CTNNB1* mutated adenoma had similar density of *CYP11B2* expression as WT patients; however, both groups of adenoma had less *CYP11B2* expression than those with *KCNJ5* mutations (all *p* < 0.01; Fig. 2a). However, there was no difference in *CYP11B1* mRNA expression levels among the three groups. (Fig. 2b). *VSNL1* and *CYP11B2* are overexpressed in APAs compared with normal adrenals, especially in those with *KCNJ5* mutation in Western blots (Fig. 3). Cyclin D1 expression was high in both *CTNNB1* and *KCNJ5* mutations. However, the GATA4 expression was not predominant in those with *CTNNB1* mutation. *CYP11B2* expressed diffusely on adenomas harboring *CTNNB1* mutations and showed mottled staining on adenomas harboring *KCNJ5* mutations (Fig. 4).

#### Histologic expression of investigated APA

Adenoma harboring *CTNNB1* mutations displayed heterogeneous cytoplasmic, membranous and nuclear expression of active β–catenin. The *CTNNB1* mutants displayed higher, diffuse active β–catenin expression than KCNJ5 mutation carriers or WT, especially in adenomas from female patients; and showed prominent nuclear staining. Adenomas with mutant *CTNNB1* and all investigated adenomas revealed an unremarkable expression of GATA4.

GnRHR showed diffuse cytoplasmic, membranous and nuclear expressions on adenomas, and was especially enhanced in adenoma harboring *CTNNB1* mutations from female patients. GnRHR was attenuated in *KCNJ5* mutated adenomas. LHCGR was diffusely expressed in adrenal tissues and was prominent in adenoma harboring *CTNNB1* mutations. The expression of GnRHR were non-specifically and diffusely stained both in areas with *CYP11B1* and *CYP11B2* expression (Fig. 4).

## Discussion

About 3.7% (8 adenomas) of our 219 APA patients were found to harbor somatic *CTNNB1* mutations, and their molecular expressions and clinical outcomes were reported. This low prevalence is similar to the 5.1% (10/189) reported in APAs^29^, but much lower than those reported in 15–26.9% of various types of adrenal adenomas and up to 30.8% of adrenocortical carcinomas^30^,^31^.

Of great interest, we found that patients who harbor *CTNNB1* mutations had a higher likelihood of residual hypertension after adrenalectomy, when compared with wild-type APA patients or *KNCJ5* mutation carriers. This is in contrast to the first case report of *CTNNB1* mutation in a female APA patient who had her hypertension cured after adrenalectomy^10^. Although *CTNNB1* mutation carriers had a shorter duration of hypertension, their average age was higher than the other groups. One of the possible explanations of the higher post-adrenalectomy residual hypertension among the patients who harbor *CTNNB1* mutations could be that age-related essential hypertension played an important role in hypertension observed for these patients. Their shorter hypertensive latency and older ages could indicate that their hypertension is not only a reflection of the severity of excessive aldosterone related vascular remodeling. Several other mechanisms could explain residual hypertension after adrenalectomy, such as vascular damage, endothelial dysfunction, and arteriolosclerosis^32^^,46^. Age could give a high predictive power and represents a significant independent risk factor for effecting hypertension cure rate^33^. Future studies are needed to determine the long-term cardio-vascular events in patients with or without mutations, especially focusing on the effects of variable somatic mutations.

Our study found that not only were *CTNNB1* mutations more prevalent in women with APA^29^, but also that these women were diagnosed at their menopausal or postmenopausal ages. This is in contrast to a previous study, where female patients were identified during pregnancy or during childbearing age. We performed a literature systemic review of all *CTNNB1* mutations in adrenal APAs and showed that out of 16 cases formerly reported and our 8 cases described herein, 75% of them were women^3^,^10^,^29^,^30^,^34^.

In addition to confirming the previous reports which showed that APAs harboring *CTNNB1* mutation could display *CYP11B1* or *CYP11B2* heterogeneous expression^29^, or in both CYP11B2-positive and CYP11B2-negative regions^35^; our results showed that somatic *CTNNB1* mutations in APAs were not only observed in both CYP11B2-positive and CYP11B2-negative regions, but also existed in CYP11B1-positive areas. Diverse staining of CYP11B1 and CYP11B2 were previously documented only in 2 cases^29^ and here we further demonstrated these results in our 6 female cases, with a total of 8 cases to reinforce this finding. All these findings, together with the reported higher prevalence of *CTNNB1* mutations among other adrenal adenomas^30^ and adrenal cancers^36^, suggest that *CTNNB1* mutations may be more related to tumor cell growth (tumorigenesis), rather than to actual aldosterone production. It is also consistent with our result that Wnt/β-catenin pathway drives down-streamed cyclin D1 transcription, a gene involved in cell growth^37^ in adenomas with *CTNNB1* mutations compared with wild-type APA adenomas.

We observed the existence of *CTNNB1* mutations in APAs seemed mutually exclusive to the mutations in *KCNJ5, ATP1A1* and *ATP2B3*. This might indicate the possibility that aberrantly activated β-catenin signals for adrenal tumor formation^8^. Recently, activated Wnt/β-catenin signaling has been reported in 70% of APAs and as a contributor to adrenal tumorigenesis^6^. In APAs harboring *CTNNB1* mutations, the nuclear and/or cytoplasmic accumulation of active β-catenin protein increased, especially for female patients. It is proposed that *CTNNB1* mutations stabilize β-catenin and increase the activity of the finely tuned Wnt signaling pathway, leading to tumor formation^38^. The positive nuclear β-catenin staining indicates the active components of Wnt/β-catenin signaling could lead to β-catenin protein accumulation^29^,^39^. The accumulation of β-catenin protein and increased expression of cyclin D1, VSNL1 and the aberrantly active Wnt signaling could be involved in APA proliferation and anti-apoptosis^23^,^24^.

Most of our *CTNNB1* mutations were identified at S45P (62.5%), which is slightly lower than previously reported (80%)^29^. Phosphorylation with GSK-3 regulates β-catenin degradation and mutations with altered serine/threonine residues in the GSK-3 binding domain decrease β–catenin degradation^39^.

Although not statistically significant, there was a trend for the APA patients harboring *CTNNB1* mutations to have larger tumor sizes, higher serum potassium and creatinine levels, which implicate that there could be other factors than just aldosterone alone to affect the underlying etiologies and severity of hypertension among these patients. This finding is different to a previous report where patients harboring *CTNNB1* mutations had larger adenomas but did not have higher aldosterone compared to patients with no mutations^29^.

Constitutive activation of the Wnt signaling pathway in zona glomerulosa-like adenomatous cells could lead to de-differentiation toward their common adrenal-gonadal precursor cell type, and lead to aberrant expression of gonadal receptor LHCGR and/or GnRHR^10^. In a subset of non-pregnant PA patients from our cohort, the aberrant GnRHR was expressed and several of these patients had increased aldosterone secretion^40^. The GnRHR staining identified in normal and APA adrenal tissues in this study was consistent with previous reports^41^. Although there was increased expression of GnRHR and LHCGR in wild-type APA patients and less in *KCNJ5* mutation adenoma^42^, we further showed patients harboring *CTNNB1* mutations over-expressed GnRHR and LHCGR compared adenomas with *KCNJ5* mutation in both genders. Prior studies have reported the over-expression of GnRHR (55%) and LHCGR (41.7%) in APAs, however the status of *CTNNB1* was not evaluated^42^,^43^. The over-expression of GnRHR and LHCGR in a high proportion of APAs is probably a consequence of events other than an activating mutation in *CTNNB1*^44^. Interestingly, we observed that *CTNNB1* mutated APAs with diffuse GnRHR expression occurred both in areas with CYP11B1 and CYP11B2 expression. As mentioned earlier, all six of our female patients with *CTNNB1* mutated APAs were discovered either at menopause or postmenopausal ages. Although gonadotropin-releasing hormone, through stimulated GnRHR might regulate aldosterone production in rare cases of APA^43^,^44^, most APA patients with *CTNNB1* mutations were not identified during pregnancy.

Our findings also confirm that there is high prevalence of *KCNJ5* mutations among APA patients in Taiwan, and the prevalence of APA tumors harboring other specific mutations (e.g. *ATP, CACNA, CTNNB1*) is considerably low^18^,^43^,^45^. The current cohort and others from Asia (ranging from 59.5 to 76.8%)^18^,^43^ reported a higher prevalence of APAs harboring *KCNJ5* somatic mutations (52.9% in this cohort). This finding differs to related reports from Western countries (ranging from 12.5 to 46.3% of *KCNJ5* mutations among APAs)^32^, and might suggest the presence of certain genetic and epidemiological differences between Asian and Western populations.

There were some strengths and limitations to our study. Using the standard diagnostic implementation criteria and with patients possessing the same ethnic background, enrollment and sample collection for this study was standardized and unified across all participating centers. Under the standard methods among the hypertensive patients evaluated, the higher rate of *KCNJ5* somatic mutations for APAs is unlikely to be related to differences in diagnosis and treatment methods^18^.

Searching for further “sleeper or dormant” somatic mutations that are silent until triggered by some specific identifiable events could shed more light on the study of APAs in the future^18^,^29^. Although unilateral adrenalectomy represents the treatment of choice for lateralized PA, further investigations on somatic mutations of APAs may disclose some interesting new drug targets for some subgroups of APAs not eligible or not amenable for surgery, especially in the area of higher prevalence of somatic mutation-carriers. Further studies to identify these somatic mutation patients without analysis of the tumor DNA specimen is challenging, but could save these patients from undergoing surgery with substantial long-term benefits.

## Conclusions

In summary, we described *CTNNB1* somatic mutation prevalence among our APA patients, along with its phenotype and clinical outcomes, and identified a female gender dominance and higher risk for post-adrenalectomy residual hypertension. APAs harboring *CTNNB1* mutations have variable expressions of CYP11B1 and CYP11B2, and heterogeneous expressions of gonadal receptors. All these points suggest the possibility that *CTNNB1* mutations in APAs may be more related to tumorigenesis rather than aldosterone production.

## Additional Information

**How to cite this article**: Wu, V.-C. *et al*. The prevalence of CTNNB1 mutations in primary aldosteronism and consequences for clinical outcomes. *Sci. Rep.* **7**, 39121; doi: 10.1038/srep39121 (2017).

**Publisher's note:** Springer Nature remains neutral with regard to jurisdictional claims in published maps and institutional affiliations.

## Supplementary Material

Supplementary Information

## Figures and Tables

**Table 1:** The clinical characteristics of *CTNNB1* mutations.

Pt ID	Mutation	Sex	Age	APA diameter (mm)	Side	AVS	NP59	Number of nodules	Aldosterone (ng/dL)	Renin (ng/mL/hr)	Potassium mmol/dL	Categories before OP*	Categories after OP*
Pt1	S45F	F	50	1.6	L	lateralization	—	1	28.9	0.31	3.7	1	1
Pt2	S45P	F	54	1.0	R	lateralization	—	1	26.1	0.04	4.2	2	1
Pt3	S45P	F	51	1.0	L	lateralization	lateralization	1	86.0	0.34	4.5	3	0
Pt4	S45P	F	86	2.1	R	lateralization	lateralization	1	27.7	0.09	3.2	3	2
Pt5	S45P	M	62	3.0	R	—	lateralization	1	40.5	0.27	3.7	3	3
Pt6	S45P	F	58	1.2	L	lateralization	lateralization	1	195.8	0.24	3.4	3	2
Pt7	S45F	F	62	2.7	R	lateralization	—	2	22.1	0.66	4.1	1	1
Pt8	S45F	M	67	4.3	R	—	lateralization	1	24.8	0.35	4.0	1	1

^*^Categories of antihypertensive drugs.

Abbreviations: APA, aldosterone-producing adenoma; AVS, adrenal venous sampling; F, female,; M, male; L, left, OP, operation; Pt, patietns; R, right. NP-59 (SPECT/CT), I131–6b-iodomethyl-19-norcholesterol/SPECT/CT.

**Table 2:** Clinical and biochemical characteristics of study patients during screening.

	*CTNNB1*(n = 8)	Before matching	WT*(n* = *89)*	*p*§	*p*^*2*^	After matching	WT (n = 24)	*p*§	*p*∥
		*KCNJ5*(n = 116)				*KCNJ5*(n = 24)			
Gender, male (%)	2 (25)	48 (41.4)	45 (50.6)	**0.302**	**0.155**	7 (29.2)	12 (50)	**0.602**	**0.207**
Age (years)	60.4 ± 8.7	46.4 ±10.6	55.8 ± 11.1	**<0.001**	**0.261**	56.1 ± 4.5	60.2 ± 6.0	**0.078**	**0.946**
MBP (mmHg)	101.1 ± 14.3	110.9 ± 20.3	107.6 ± 18.1	**0.213**	**0.355**	110.5 ± 13.0	105.7 ± 14.2	**0.111**	**0.454**
Tumor size (cm)	2.1 ± 1.2	1.7 ± 0.5	1.7 ± 0.7	**0.076**	**0.162**	1.7 ± 0.6	1.7 ± 0.7	**0.072**	**0.185**
Duration of HTN (years)	1.1 ± 2.1	5.9 ± 5.4	8.7 ± 8.7	**<0.001**	**<0.001**	10.2 ± **5.2**	10.3 ± 8.1	**<0.001**	**<0.001**
Family history of HTN (%)	2 (25)	57 (49.4)	40 (44.9)	**0.278**	**0.676**	14 (58.3)	10 (41.7)	**0.220**	**0.676**
BMI (kg/m^2^)	24.1 ± 3.5	25.2 ± 4.3	25.3 ± 03.9	**0.515**	**0.450**	24.74 ± 3.1	24.3 ± 2.8	**0.675**	**0.888**
Diabetes (%)	1 (12.5)	11 (9.6)	20 (22.5)	**0.571**	**0.448**	3 (12.5)	4 (16.7)	**0.705**	**0.633**
Proteinuria (%)	5 (62.5)	55 (47.4)	35 (39.3)	**0.477**	**0.268**	11 (45.8)	9 (37.5)	**0.685**	**0.412**
Smoker (%)	0 (0)	19 (16.5)	14 (15.7)	**0.250**	**0.273**	5 (20.5)	3 (12.5)	**0.211**	**0.408**
Serum K (mmol/L)	3.9 ± 0.4	3.1 ± 0.6	3.7 ± 0.5	**<0.001**	**0.432**	3.2 ± 0.6	3.7 ± 0.4	**0.009**	**0.359**
Serum Cre (mg/ dL)	1.28 ± 1.20	0.9 ± 0.2	0.97 ± 0.35	**0.007**	**0.081**	0.95 ± 0.29	0.92 ± 0.19	**0.223**	**0.155**
PH	7.41± 0.03	7.44 ± 0.04	7.41 ± 0.05	**0.255**	**0.944**	7.44 ± 0.03	7.40 ± 0.05	**0.175**	**0.652**
HCO3^-^	24.9 ± 3.3	25.8 ± 6.0	24.3 ± 4.3	**0.165**	**0.581**	25.1 ± 3.5	22.7 ± 3.6	**0.305**	**0.929**
Log PAC (ng/dL)	1.59 ± 0.35	1.73 ± 0.30	1.62 ± 0.30	**0.197**	**0.807**	1.70 ± 0.27	1.56 ± 0.25	**0.371**	**0.817**
PRA (ng/mL/hr)	0.29 ± 0.19	0.42 ± 0.77	0.98 ± 2.34	**0.633**	**0.403**	0.19 ± 0.2	1.1 ± 3.8	**0.231**	**0.557**
Log ARR (ng/dL per (ng/mL/hr)	2.24 ± 0.50	2.56 ± 0.77	2.19 ± 0.75	**0.248**	**0.829**	2.68 ± 0.74	2.32 ± 0.83	**0.136**	**0.823**
Hypertensive agents at screening									
a-blocker	3 (62.5)	31 (26.7)	16 (18)	**0.683**	**0.187**	10 (41.7)	4 (16.7)	**0.999**	**0.327**
β- blocker	2 (25)	40 (34.5)	34 (38.2)	**0.715**	**0.171**	16 (66.7)	11 (45.8)	**0.999**	**0.420**
Calcium channel blocker	6 (75)	89 (76.7)	54 (60.7)	**0.999**	**0.706**	16 (66.7)	17 (70.8)	**0.999**	**0.999**
Vasodilator	2 (25)	4 (4.3)	5 (5.6)	**0.065**	**0.102**	0 (0)	3 (12.5)	**0.056**	**0.578**
ACEI/ARB	3 (62.5)	44 (37.9)	44 (49.4)	**0.999**	**0.716**	7 (29.2)	11 (45.8)	**0.681**	**0.999**
Recovery of hypertension	1 (12.5)	92 (79.3)	51 (57.3)	**<0.001**	**0.018**	16 (66.7)	13 (54.2)	**0.011**	**0.047**

^§^KCNJ5 vs CTNNB1.

^∥^WT vs. CTNNB1.

Abbreviations: ACEI/ARB, Angiotensin Converting Enzyme Inhibitors/Angiotensin Receptor Blockers. ARR, aldosterone to renin ratio (ng/dL per ng/mL/hr); APA, aldosterone producing adenoma; eGFR, estimated glomerular filtration rate; EH, essential hypertension; K, potassium; MBP, mean blood pressure; PAC, plasma aldosterone concentration; PRA, plasma renin activity. Data are shown as the mean values ± standard deviation. Note: To convert potassium in mmol/L to mEq/L, multiply by 1; PAC in ng/dL to nmol/L, multiply by 0.02774; PRA in ng/mL/hr to ng/L/s, multiply by 0.2778; ARC in ng/dL to pmol/L, multiply by 0.0361.

**Table 3:** Factors associated with post-operative residual hypertension#.

	Before matching*	lower 95% CI	*p*	After matching**	95% CI	*p*
	HR			HR		
Age (per year)	1.03	1.00–1.06	0.049*	**1.01**	**0.85–1.19**	**0.947**
Sex (male)	1.24	0.57–2.73	0.590	**5.23**	**1.46–18.8**	0.011*
Duration of HTN (years)	1.01	0.96–1.07	0.621	1.13	0.98–1.30	0.089
Family history of HTN	0.89	0.45–1.74	0.725	0.38	0.06–2.32	0.296
Tumor size (cm)	0.91	0.54–1.51	0.711	0.39	0.10–1.57	0.184
Smoking (yes)	1.61	0.63–4.10	0.318	3.04	0.22–42.3	0.408
BMI (kg/m^2^)	1.16	1.07–1.26	<0.001*	1.54	1.10–2.17	0.014*
MBP (mmHg)	1.01	0.99–1.03	0.313	0.99	0.93–1.06	0.835
Diabetes Mellitus (yes)	1.83	0.75–4.48	0.185	0.48	0.06–4.10	0.505
Log ARR (ng/dL per (ng/mL/hr)	0.69	0.439–1.09	0.113	0.24	0.05–1.01	0.051
potassium (mmol/L)	1.36	0.74–2.49	0.322	1.51	0.19–11.75	0.697
Cre (mg/dL)	1.13	0.39–3.29	0.822	1.12	0.12–10.8	0.922
Mutation						
*CTNNB1* vs *KCNJ5*	18.9	1.99–166.7	0.010*	18.2	1.72–200	0.016*
*CTNNB1* vs WT	9.1	0.88–71.4	0.051	14.5	1.33–166.7	0.028*

^#^Logistic regression analysis with a stepwise variable selection procedure was applied using available variables (listed in Table 2) to identify important factors associated with post-operative residual hypertension.

Multiple logistic regression model before matching*: adjusted generalized R^2^ = 0.290, and Hosmer-Lemeshow goodness-of-fit test *p *= 0.536 (degree of freedom = 8). After matching**: adjusted generalized R^2^ = 0.564, and Hosmer-Lemeshow goodness-of-fit test *p* = 0.620 (degrees of freedom = 7). Abbreviations: APA, aldosterone-producing adenoma; ARR, aldosterone to renin ratio; BMI, body mass index; Cre, creatinine; EH, essential hypertension; HR, hazard ratio; mBP, mean blood pressure.
