# Supplementary material for: The prevalence of CTNNB1 mutations in primary aldosteronism and consequences for clinical outcomes
Source: Sci Rep. 2017 Jan 19;7:39121. doi: 10.1038/srep39121 (PMC5244399; doi:10.1038/srep39121)
Supplement: Supplementary Information [file srep39121-s1.doc]

**Title:**

The prevalence of CTNNB1 mutations in primary aldosteronism and consequences for clinical outcomes

**Authors:**

Vin-Cent Wu, Shuo-Meng Wang, [Shih-Chieh Jeff Chueh](http://jra.sagepub.com/search?author1=Shih-Chieh+Jeff+Chueh&sortspec=date&submit=Submit), Shao-Yu Yang, Kuo-How Huang, Yen-Hung Lin, Jian-Jhong Wang, Rory Connolly, Ya-Hui Hu, Celso E. Gomez-Sanchez , Kang-Yung Peng, Kwan-Dun Wu

***Supplementary Material and methods***

**Patients were enrolled from the following hospitals:**

This study included two medical centers (National Taiwan University Hospital (NTUH), Taipei, Taiwan; Taipei University Hospital, Taipei, Taiwan) and five regional hospitals (Cardinal Tien Hospital, New Taipei City, Taiwan; Taipei Tzu Chi Hospital, New Taipei City, Taiwan; Yun- Lin Branch of NTUH, Douliou City, Taiwan; Hsin-Chu Branch of NTUH, Hsin-Chu City, Taiwan; Zhongxing Branch of Taipei City Hospital, Taipei, Taiwan).

**Our standard protocol to identify primary aldosteronism (PA) and functional laterization:**

The diagnosis of primary aldosteronism was established in hypertensive patients on the basis of the following criteria [^1-4^](#_ENREF_1)(Fig S1):

***Confirmation***

Fulfillment of the following three conditions confirmed a diagnosis of PA:

(1) autonomous excess aldosterone production evidenced with an aldosterone-renin ratio (ARR) > 35; (2) a TAIPAI score larger than 60% [^5^](#_ENREF_5); (3) post-saline loading PAC > 10 ng/dL, or PAC/PRA > 35 (ng/dL)/(ng/mL/h) shown in a post-capotopril/losartan test, or PAC > 6 ng/dL indicated by a fludrocortisone suppression test [^4^](#_ENREF_4). (Abbreviations: PAC, plasma aldosterone concentration; PRA, plasma renin activity).

***Lateralization***

1. APA was identified on the basis on the following four criteria: (1) autonomous excess aldosterone production evidenced with an ARR > 35, a TAIPAI score larger than 60% [^5^](#_ENREF_5), and post-saline loading PAC > 10 ng/dL; (2) adenoma evidenced with a CT scan for pre-operative evaluation [6]; (3) lateralization of aldosterone secretion at adrenal vein sampling (AVS) or during dexamethasone suppression NP-59 SPECT/CT [^6^](#_ENREF_6); (4) pathologically proven adenoma after an adrenalectomy for those who underwent operations, and subsequent emergence of either a cure pattern of hypertension without anti-hypertensive agents or improvement in hypertension, potassium, PAC, and PRA .
2. Idiopathic hyperaldosteronism (IHA) was distinguished on the basis of the following four criteria: (1) autonomous excess aldosterone production evidenced with an ARR > 35, a TAIPAI score larger than 60% [^5^](#_ENREF_5), and post-saline loading PAC > 10 ng/dL; (2) evidence of bilateral diffuse enlargement indicated by a CT scan for pre-operative evaluation; (3) non-lateralization of aldosterone secretion at AVS or during dexamethasone suppression NP-59 SPECT/CT [^6^](#_ENREF_6); (4) evidence of diffuse cell hyperplasia reported in follow-up pathology studies for those with operations.

**References**

1. Sechi, L.A.*, et al.* Long-term renal outcomes in patients with primary aldosteronism. *Jama* **295**, 2638-2645 (2006).

2. Wu, V.C.*, et al.* Primary Aldosteronism: Diagnostic Accuracy of the Losartan and Captopril Tests. *Am J Hypertens* **22**, 821-827 (2009).

3. Kuo, C.C.*, et al.* Verification and evaluation of aldosteronism demographics in the Taiwan Primary Aldosteronism Investigation Group (TAIPAI Group). *J Renin Angiotensin Aldosterone Syst* **12**, 348-357 (2011).

4. Chao, C.T.*, et al.* Diagnosis and management of primary aldosteronism: an updated review. *Annals of medicine* **45**, 375-383 (2013).

5. Wu, V.C.*, et al.* Kidney impairment in primary aldosteronism. *Clin Chim Acta* **412**, 1319-1325 (2011).

6. Yen, R.F.*, et al.* 131I-6beta-iodomethyl-19-norcholesterol SPECT/CT for primary aldosteronism patients with inconclusive adrenal venous sampling and CT results. *J Nucl Med* **50**, 1631-1637 (2009).

7. Wu, V.C.*, et al.* Diagnosis and Management of Primary Aldosteronism. *Acta Nephrologica* **26**, 111-120 (2012).

Table S1. Primer sequences

| **Primer name** | **Primer sequence (5’→3’)** |
| --- | --- |
| **KCNJ5_1F** | GATGGTGTCTTTTTAACTCAAAGC |
| **KCNJ5_1R** | GTGATGACTCGGAAGCCATACC |
| **KCNJ5_2F** | CTTTCCTGTTCTCCATTGAGACC |
| **KCNJ5_2R** | CTGAGGAGGACAAAGCGCC |
| **KCNJ5_3F** | ATGCATGTAACTTCCGTTTCCC |
| **KCNJ5_3R** | GCCAGTGACAGGAGGTCTTAGG |
| **KCNJ5_4F** | CTTCATTTGGTGGCTCATTGC |
| **KCNJ5_4R** | GGGACTTGATGAGCTTGGC |
| **ATP1A1_4F** | TTCCTTGGGCCTATTGTTTG |
| **ATP1A1_4F** | GTGGGAGACAAAGACGGAGA |
| **ATP1A1_8F** | CGTGGCTTCCTTCAGGTTAG |
| **ATP1A1_8R** | CGTGATGTGGCTCTCAAGAA |
| **ATP2B3_8F** | CCTGGGCTGTTTATCCTGAA |
| **ATP2B3_8R** | CCCCA GTTTC CGAGT CTGTA |
| **CACNA1D_8aF** | AGCTGCAACTGGGGCTC |
| **CACNA1D_8aR** | GCAGCTAGGAGACACGCAG |
| **CACNA1D_17F** | TTTACTTCTGTAGACTGTCCTTTTA |
| **CACNA1D_17F** | ACACGTGACTCCCACTCTCAGC |
| **CACNA1D_6F** | GTAAAGGAGGCATGGTTAGG |
| **CACNA1D_6R** | TGGCTCAGTAAATGTGCTGGT |
| **CACNA1D_8bF** | GCCTTGATGACTCTGTGTG |
| **CACNA1D_8bR** | CCAGCAAAGCTTGTGTGGT |
| **CACNA1D_23F** | CACGCTAACTGTGCAGGGA |
| **CACNA1D_23R** | TCAGCTCTGCCCAGAAGAG |
| **CACNA1D_27F** | CCAATCTACAACCACCGCGT |
| **CACNA1D_27R** | GACCAAGGGACAGAAGCCAA |
| **CACNA1D_32F** | ACGGTTCTTCCTCACTGTCG |
| **CACNA1D_32R** | CTTCAGCAGAGGCATTTGGCT |
| **hLHCGR_81F** | TCTGG AGAAG ATGCA CAATG GA |
| **hLHCGR_81R** | GCCTG CAATT TGGTG GAAGA |
| **hGNRHR_62F** | GGACC GCTCC CTGGC TAT |
| **hGNRHR_62R** | ACTGT CCGAC TTTGC TGTTG CT |
| **hGATA4_68F** | TCCGT GTCCC AGACG TTCTC |
| **hGATA4_68R** | GAGAG GACAG GGTGG ATGGA |
| **CTNNB1_3F** | CATTC TGCTT TTCTT GGCTG TC |
| **CTNNB1_3R** | GCTAT TACTC TCTTT TCTTC ACC |

**Figure S1**. The subtype-differentiating protocol of the TAIPAI group .


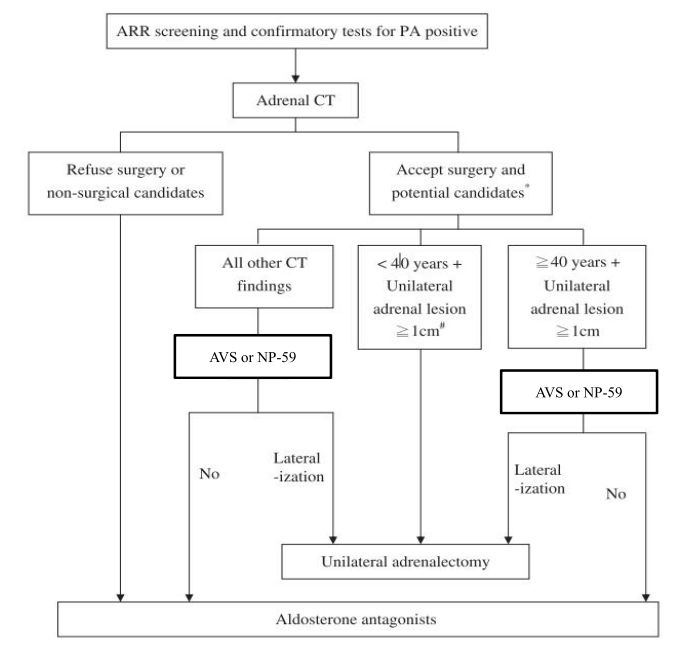


* Abbreviations: AVS, adrenal venous sampling; APA, aldosterone-producing adenomas; IHA, idiopathic hyperaldosteronism; NP-59 (SPECT/CT), I131-6b-iodomethyl-19-norcholesterol/SPECT/CT; INC, incidentaloma.

**Figure S2**. Electropherogram showing representative traces of mutations found in CTNNB1 (S45F, S45P) by Sanger sequencing of APAs.


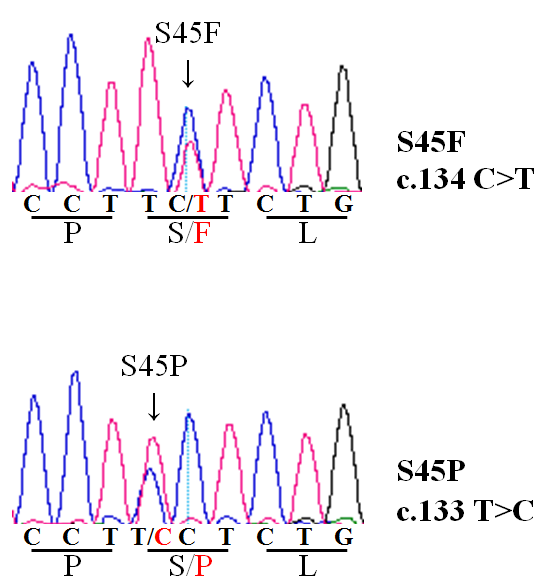


**Figure S3**. *CTNNB1* exon 3 amplification from genomic DNA of APA (size 483bp)


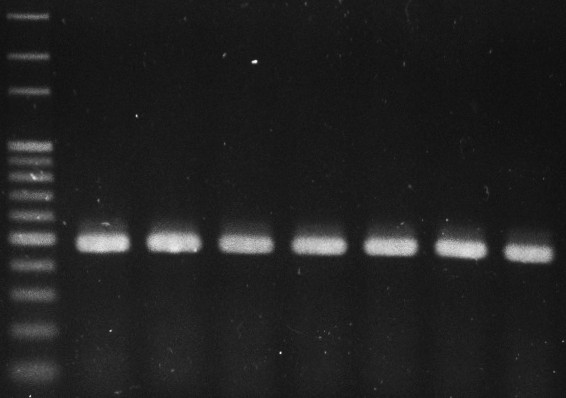


500 bp

Forward: CATTC TGCTT TTCTT GGCTG TC

Reverse: GCTAT TACTC TCTTT TCTTC ACC

**Figure S4**. Flow diagram for selection of study subjects.

**Abbreviations:** APA, aldosterone producing adenoma; AVS, adrenal venous sampling; GRA, glucorticoid remediable aldosteronism; NP-59 (SPECT/CT), I131-6b-iodomethyl-19-norcholesterol/SPECT/CT; WT, wild type.

fig1


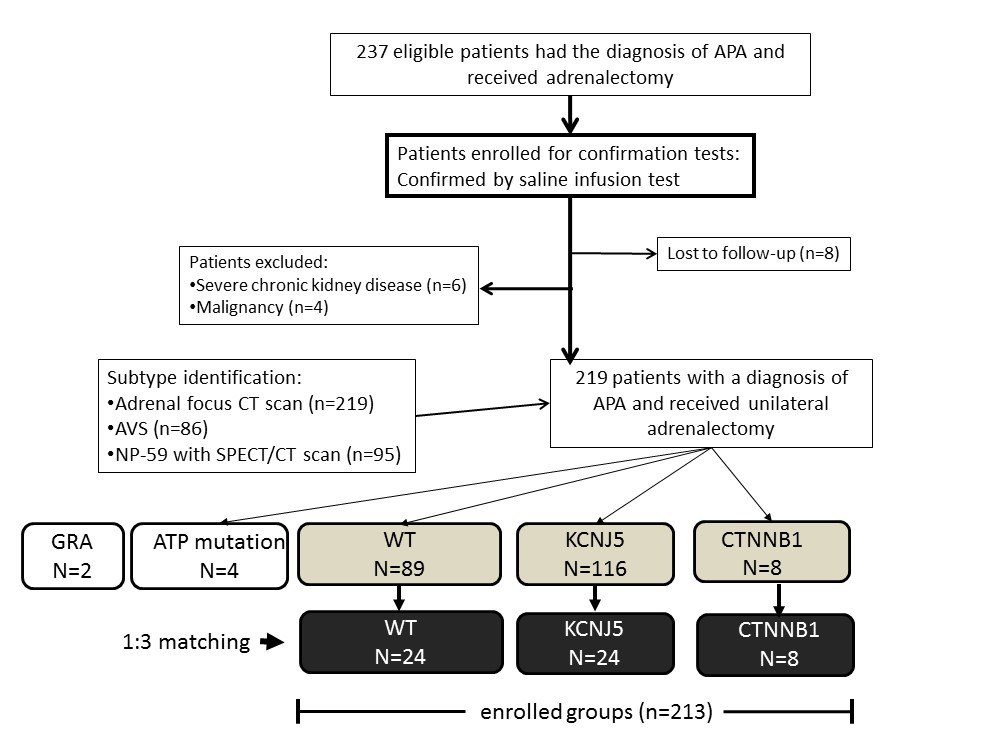


Membership of the Taiwan Primary Aldosteronism Investigation (TAIPAI) Study Group: Che-Hsiung Wu, Ya-Hui Hu, MD(Chi-Taz, PI of Committee); Vin-Cent Wu, MD (NTUH, PI of Committee); Yen-Hung Lin, MD (NTUH, PI of Committee); Yi-Luwn Ho, MD, PhD (NTUH, PI of Committee); Hung-Wei Chang, MD, PhD (Far eastern hospital, PI of Committee); Lian-Yu Lin MD, PhD (NTUH, PI of Committee); Jian-Jhong Wang MD (Chi-Mei, PI of Committee), Fu-Chang Hu, MS, ScD, Harvard statitics, Site Investigator); Kao-Lang Liu, MD (NTUH, PI of Committee); Shuo-Meng Wang, MD (NTUH, PI of Committee); Kuo-How Huang, MD (NTUH, PI of Committee); Yung-Ming Chen, MD (Yun-Lin Branch, NTUH, PI of Committee); Chin-Chi Kuo; MD (Yun-Lin, PI of Committee), Chin-Chen Chang, MD (NTUH, PI of Committee); Shih-Chieh Chueh, MD, PhD (Cleveland Clinic, , PI of Committee); Shih-Cheng Liao, MD (NTUH, PI of Committee); Ruoh-Fang Yen, MD, PhD (NTUH, PI of Committee); and Kwan-Dun Wu, MD, PhD (NTUH, Director of Coordinating Center).
